# Assessment of thoracic volume changes after the collapse of lateral rib fractures based on chest computed tomography data: computer simulation and a multiple variable linear regression analysis

**DOI:** 10.1186/s13019-020-01213-z

**Published:** 2020-07-09

**Authors:** Xiao-Kun Chen, Yi-Jun Liu, Fu-Zheng Guo, Jiu-Xu Deng, Jian Xiong, Tian-Bing Wang, Bao-Guo Jiang

**Affiliations:** 1 Department of Orthopedic Trauma, Peking University People Hospital, No. 11 Xizhimen South Road, Beijing, 100044 P. R. China; 2 National Center for Trauma Medicine, Beijing, 100044 P. R. China

**Keywords:** Computer simulation, Stereo model, Rib fractures, Chest collapse, Chest volume, Linear regression, Computed tomography data

## Abstract

**Background:**

Chest blunt trauma (CBT) and the resultant rib fractures often lead to thoracic collapse. The purpose of this study was to explore the effect of displacement of the rib fracture and thoracic collapse on the thoracic volume by using normal chest CT data.

**Methods:**

In this retrospective study, seven consecutive normal participants were selected from our hospital between June and July 2018. Normal thoracic models were reconstructed, followed by simulation of lateral fractures through the 4th to 9th ribs under three collapse modes with 1–5 cm of collapse. The thoracic collapse models (*n* = 630) were reconstructed using 3Dmax 2014. We calculated the thoracic volume and reduction percentage for each thoracic collapse model. Linear regression-based comparisons of thoracic volume reductions were performed.

**Results:**

In all three collapse modes, the degree of the collapse was linearly correlated with the mean thoracic volume reduction. The reduction percentage in the posterior collapse mode was higher than that in the anterior collapse mode (*P* < 0.001). The largest volume reductions in the anterior, posterior, and simultaneous collapse models were in the 6th rib fracture model (*P <* 0.001), 8th rib fracture model (*P <* 0.001), and 7th rib fracture model (*P <* 0.001), respectively.

**Conclusions:**

The influences of rib fracture displacement and collapse on the thoracic volume in the 6th through 8th ribs are critical in lateral rib fractures. For patients with 6th to 8th rib fractures and posterior rib collapse, surgical intervention to restore thoracic volume may be more essential.

## Background

Chest blunt trauma (CBT) accounts for more than 15% of emergency trauma cases worldwide, and is second only to head trauma in traffic injury mortality and to head and abdomen injuries in multiple injury mortality [1, 2]. About one-third of the patients with CBT have rib fractures [3, 4]. Severe rib fractures are often associated with thoracic collapse, which can significantly reduce the thoracic volume if not corrected in time [5, 6]. Therefore, assessment of thoracic volume changes is critical for evaluation of the degree of injury and treatment decisions in patients with CBT.

Chest X-ray (CXR) and chest computed tomography (CT) are conventional imaging methods for blunt trauma evaluation [7]. CXR is the routine examination for diagnosis of CBT, while multidetector row CT scans can accurately measure the thoracic cage and thoracic volumes [8]. However, under some circumstances (e.g., remote or small community hospitals), CT examinations may not be available. Therefore, optimal utilization of the limited physical and adjuvant examination information (e.g., from CXR) to assess the thoracic volume is necessary for clinical practice in such scenarios.

Although there are some guidelines for the treatment of rib fractures, most of them are based on expert consensus or clinical experiences [9], and high-level evidence from randomized controlled trials (RCTs) is not available. One of the reasons for this may be the diversity and high mortality associated with rib fractures, which makes it difficult to conduct RCTs. Another reason is that it is difficult to obtain data for the patient’s thoracic condition before the fracture, precluding comparisons of the status before and after rib fracture. Some scholars have used computer finite element analysis of rib fractures and achieved excellent results [10]. The purpose of this study was to explore the effect of displacement of the rib fracture and thoracic collapse on the thoracic volume by using normal chest CT data.

## Methods

This was a retrospective study conducted using computer simulation and multivariate linear regression analysis to determine the effect of displacement of rib fractures and thoracic collapse on the thoracic volume on the basis of normal chest CT data. This study was approved by the Peking University Research Ethics Committee of our hospital.

### Patients

From June 2018 to July 2018, 7 consecutive participants (4 men, 3 women; age, 27–48 years; average age, 35.1 years) were selected from the physical examination center of Peking University People’s Hospital. The selection criteria included 1) normal development without chest deformity; 2) age between 18 and 65 years; 3) no smoking history; and 4) no history of chest trauma or lung or chest disease.

### Computer simulation and stereo model

**Fig. 1:**
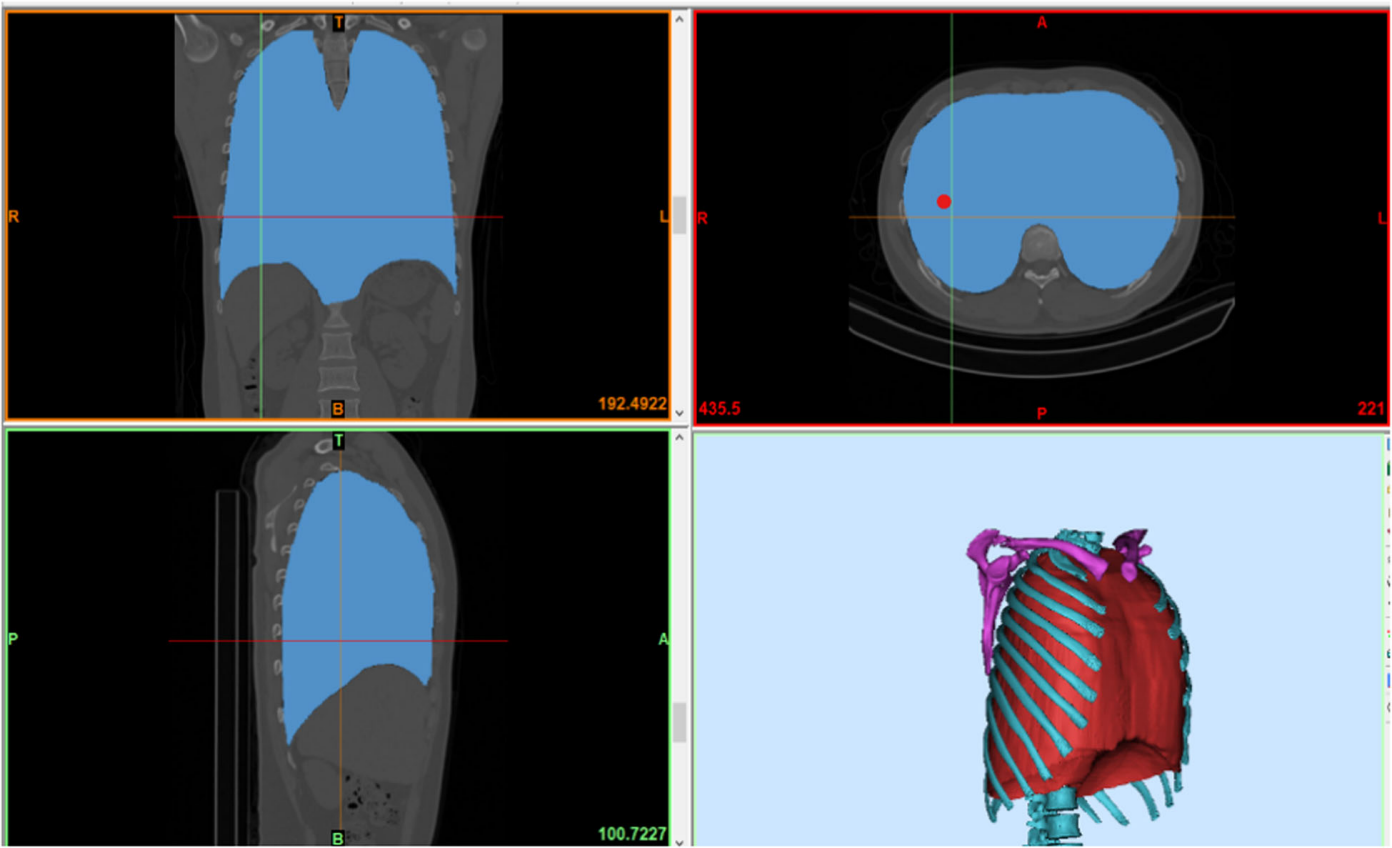
This normal thoracic model was reconstructed based on the chest CT data of a 27-year old man by using Mimics software

**Fig. 2:**
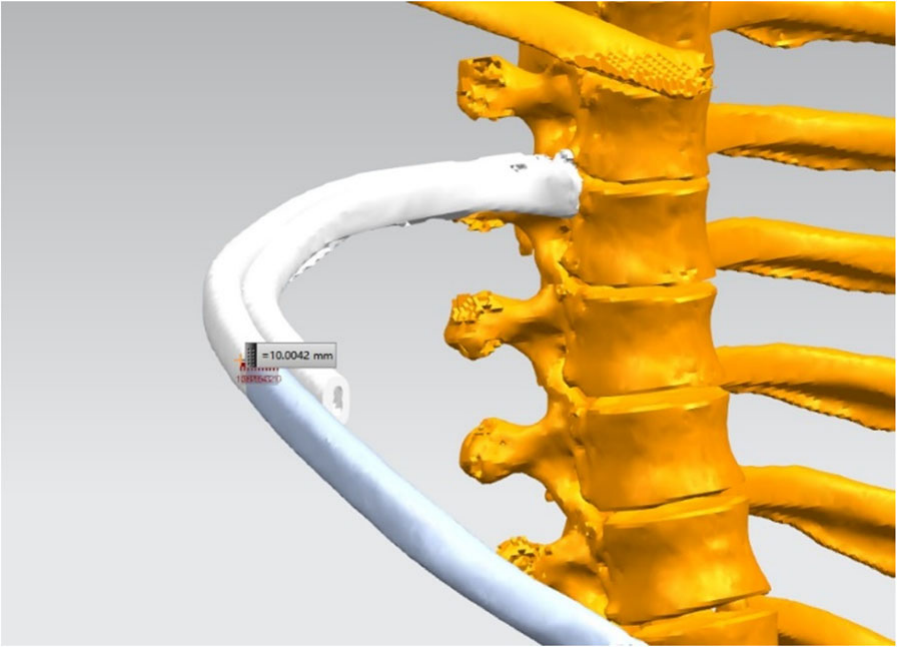
Posterior collapse mode of a lateral 6th rib fracture with a collapse degree of 1 cm

**Fig. 3:**
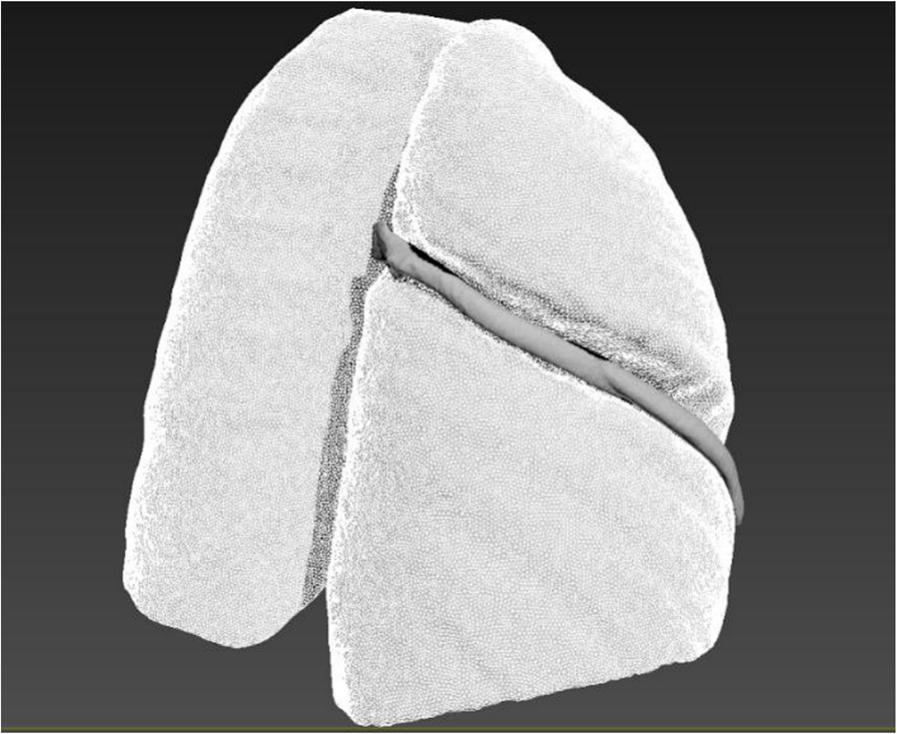
Simultaneous anterior and posterior collapse mode of a lateral 6th rib fracture with a collapse degree of 3 cm

**Fig. 4:**
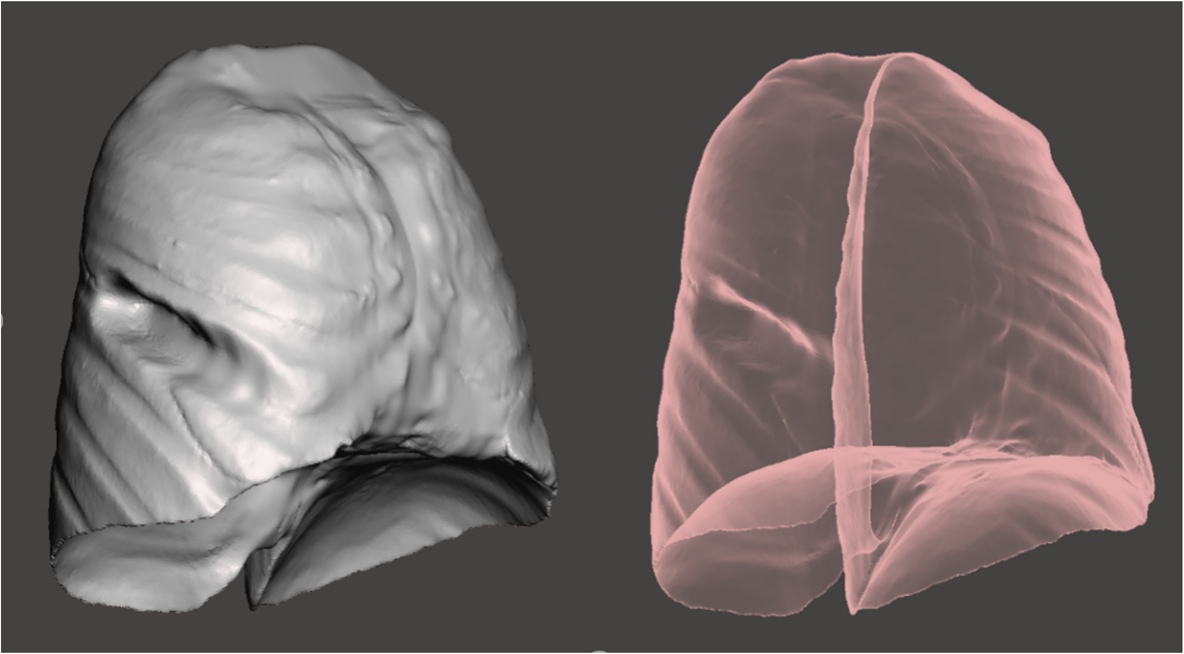
Anterior collapse mode of a lateral 7th rib fracture with a collapse degree of 4 cm

All patients underwent chest CT scans with a 64-row Siemens Somatom Definition Flash (128-slice CT). The procedure used for computer simulation and stereo modeling was as follows. First, thin-layer (≤1 mm) data were imported into Materialise Mimics 20.0 software (Materialize, Leuven, Belgium) and a three-dimensional (3D) chest model was built by using the density segmentation tool and hand-drawn selection tool (see an example of the restricted three-dimensional (3D) chest model in Fig. 1). Next, the reconstructed 3D chest model was imported into Unigraphics NX 12.0 software to simulate the lateral fractures of ribs 4 to 9 (the rib was cut along the midaxillary line) and the displacement and collapse after these fractures (see an example illustrated in Fig. 2). We simulated 3 collapse modes: posterior rib collapse (near the spine), anterior rib collapse (near the sternum), and simultaneous anterior and posterior collapse. The simulated degree of collapse ranged from 1 to 5 cm. Then, the simulated lateral rib fracture model was imported into 3Dmax 2014 software along with the previous normal thoracic model. The thoracic collapse model (see an example illustrated in Fig. 3) was simulated according to the type and degree of collapse of the rib fracture. Subsequently, the thoracic collapse model was imported into Autodesk Meshmixer software with appropriate editing to remove internal impurities such as blood vessels and bronchi, smooth the edges, and reduce the errors and facilitate thoracic volume calculation (see example illustrated in Fig. 4). Finally, the processed thoracic model and the original thoracic model were imported into the software Materialise Mimics 20.0 again, and the original normal thoracic volume, the thoracic volume of each collapse model, and the percentage reduction of the volume in each collapse model were calculated.

### Statistical methodology

A total of 630 chest fracture models of rib fractures were simulated from the 7 chest CT datasets, and the thoracic volume in each model was calculated. Multiple linear regression analysis was performed by using R (programming language). We determined the dependent variable Y as the thoracic volume, the independent variable X_1_ as the number of ribs, the independent variable X_2_ as the degree of rib collapse (centimeters), and the independent variable X_3_ as the mode of rib collapse (3 different collapse modes).

## Results

**Fig. 5:**
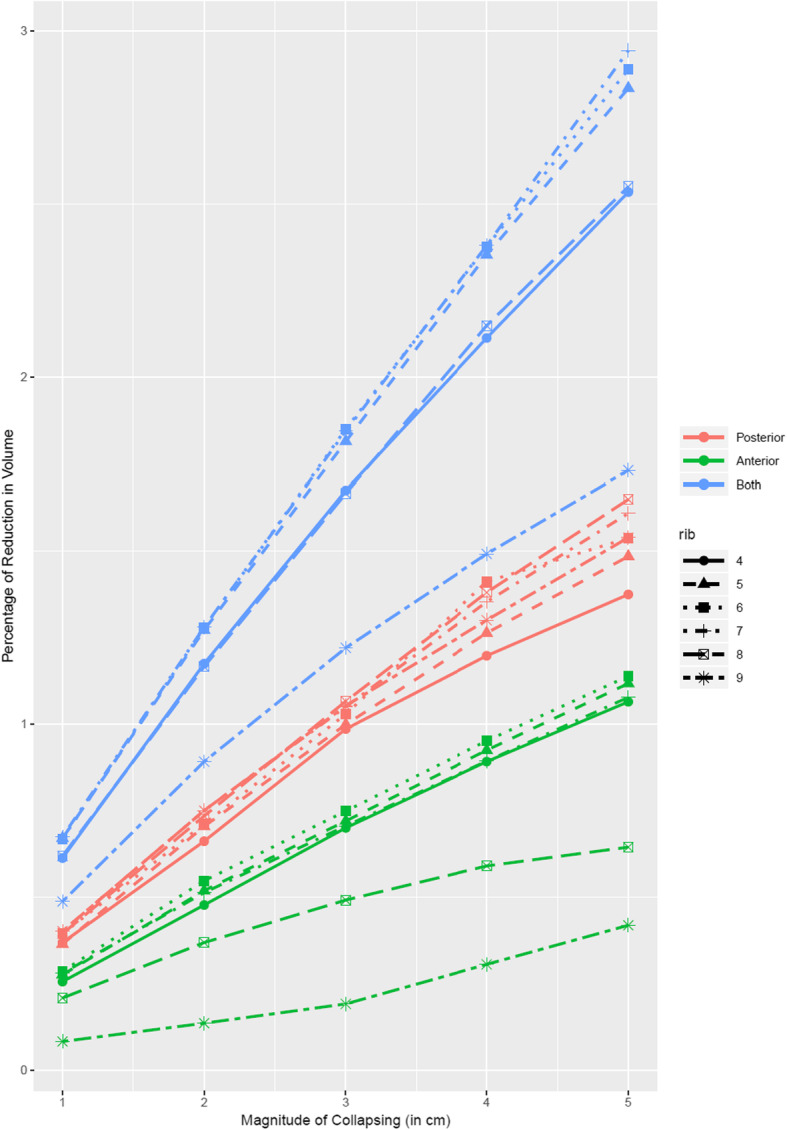
Trend of thoracic volume reduction across 18 different thoracic collapse scenarios based on 630 thoracic collapse models

In the multiple linear regression analysis, the slope prediction values for the three collapse modes were 0.29 for posterior collapse, 0.17 for anterior collapse, and 0.49 for simultaneous anterior and posterior collapse (see detailed multiple linear regression results in Appendix I). The reduction percentage of thoracic volume in the posterior collapse mode was higher than that in the anterior collapse mode (*P* < 0.001) and the reduction of thoracic volume in the simultaneous anterior and posterior collapse mode was more significant than the sum of the reductions in the anterior and posterior collapse modes at the same collapse degree (centimeters) (*P* < 0.001) (see the thoracic volume reduction trend in Fig. 5).

**Fig. 6:**
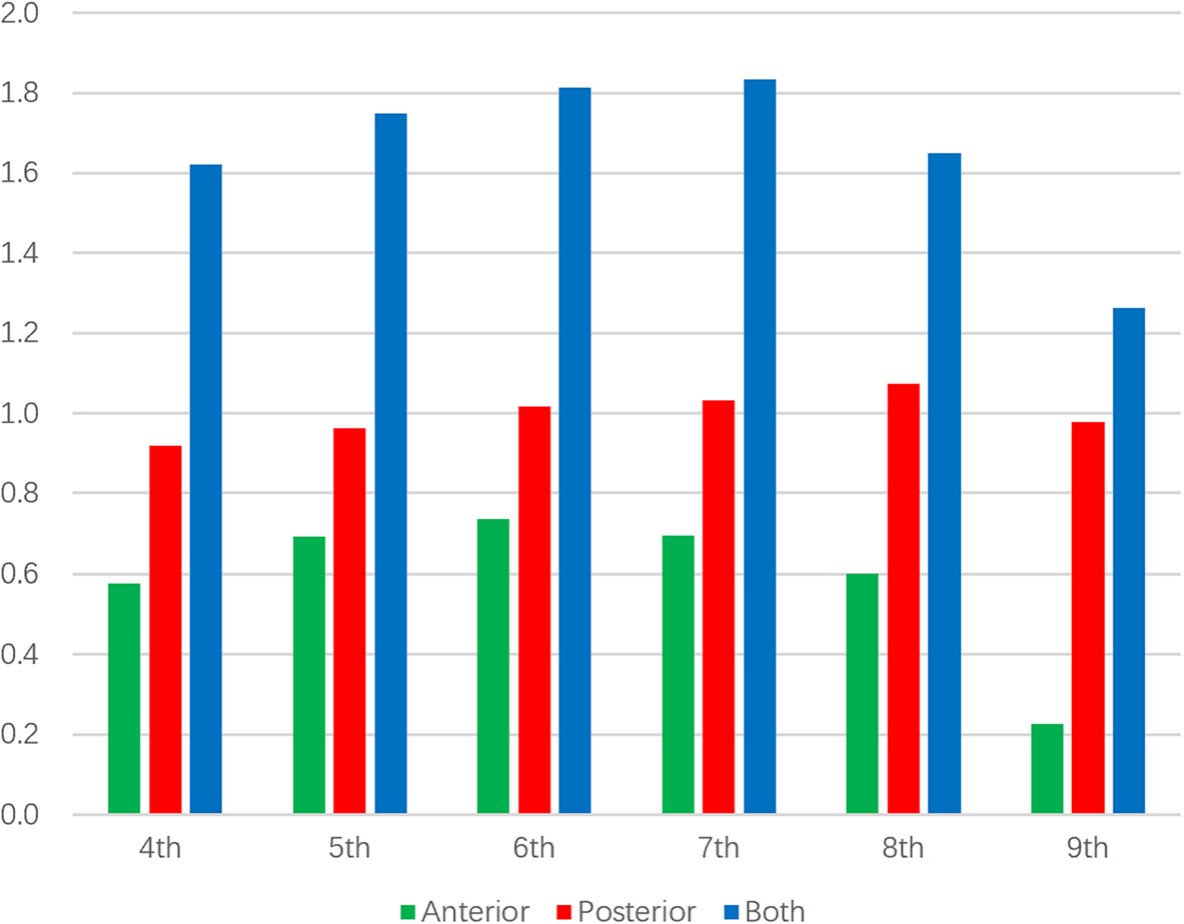
Average percentage reduction in thoracic volume in the 3 collapse modes for lateral fractures involving the 4th to 9th ribs

For the 9th lateral rib fracture model, no statistical significance was found as the percentage of thoracic volume decreased with an increase in the degree of collapse in the anterior collapse mode (*P* = 0.69). The largest volume reductions in the anterior, posterior, and simultaneous collapse modes were observed in the 6th rib fracture model (*P <* 0.001), 8th rib fracture model (*P <* 0.001), and 7th rib fracture model (*P <* 0.001), respectively. The collapse of the 4th and 5th ribs caused a relatively small reduction in the thoracic volume, while the anterior collapse of the 9th rib caused almost no change in the thoracic volume. In general, the displacement and collapse of the 6th, 7th, and 8th ribs had a key influence on the thoracic volume in side-rib fractures (see the percentage of thoracic volume reduction for each lateral rib fracture in the 3 collapse modes in Fig. 6).

## Discussion

Most CBTs occur at high speeds with the sudden deceleration of the front chest, which can cause compressive thoracic injuries. Severe thoracic deformities can affect cardiopulmonary function, reduce chest and lung compliance, and lead to impaired respiratory function. These mechanical and inflammatory sequelae often complicate the care of patients with CBT and increase the risk of pneumonia, acute respiratory failure, and even acute respiratory distress syndrome (ARDS) [11]. Patients with chest trauma and rib fractures often show respiratory dysfunction, especially when a flail chest is present. Mechanical ventilation is often needed to maintain the patient’s blood oxygen saturation [4]. Due to the chronic, and long-term pain, patients with rib fractures, cannot take deep breaths, cough, or expectorate, which can result in respiratory dysfunction [12].

The anatomical structure of the 24 ribs in the human body is complex because of the irregular shape and different lengths of each rib. As a result, when a rib fracture occurs, the location and type of fracture can be very complex [13]. Clinically, lateral rib fractures are mostly located in the 4th to 9th ribs [14]. Since ribs are flat and slender when the chest is exposed to direct or indirect trauma, the stress is concentrated at the middle part of the rib, making the sides of the ribs more prone to fracture [15]. In patients with rib fractures, the thoracic volume will decrease after the trauma. The greater the number of rib fractures, the greater is the degree of collapse, and worse is the prognosis of the patients. It was suggested that surgical stabilization of rib fractures (SSRF) should be considered in patients with multiple, severe (bicortical) displaced fractures [16]. The reduction of thoracic volume directly affects the respiratory function of the patients. In this study, we chose to study thoracic volume instead of lung volume because the thoracic volume is the factor most directly affected by rib fractures, and there are many other factors influencing lung volume, such as atelectasis and hemopneumothorax.

The results showed that the reduction in thoracic volume in the posterior collapse mode models was more obvious than that in the anterior collapse mode models (prediction slope, 0.29 > 0.17; *p <* 0.001). There are two reasons for this. One is that as the length and curvature of the rib on the posterior side are larger than those on the anterior side, when the anterior rib and the posterior rib collapse over the same distance, the posterior lateral collapse of the ribs will reduce thoracic volume to a greater degree. The second reason is that the entire shape of the thorax is narrow in the front and wide at the back, so the posterior part of lateral rib fracture collapse will reduce thoracic volume to a greater extent than the anterior part under the same conditions.

We found that the reduction of thoracic volume in the simultaneous anterior and posterior collapse model was greater than the sum of the reductions in the anterior and posterior collapse models at the same degree of collapse (centimeters) (*P* < 0.001). The reason is that when the anterior or posterior part of the rib collapses, the resultant volume reduction decreases from the fracture site to the normal end, but when both anterior and posterior parts of the rib collapse, this situation will not occur.

As shown in the results, when lateral rib fractures occur, in general, the 6th to 8th lateral rib collapse has the greatest impact on the thoracic volume. This could be because the upper part of the chest is narrow while the lower part is wide, and the cross-sectional area of the chest increases at the lower planes. The 4th and 5th ribs are located at a relatively higher position in the chest, so their fracture and collapse have less impact on the thoracic volume. Moreover, the diaphragm of normal people has an arc-shaped concave surface, in which the upper part of the diaphragm is the thorax and the lower part is the abdomen, so the anterior collapse mode in lateral fracture of the 9th rib results in a greater reduction of abdominal volume than thoracic volume. Thus, the results of simulated rib fractures may be related to the anatomical location of the ribs and the diaphragm [13].

The results of this study can be used to guide clinical treatment. The mortality rate of patients with posterior rib fracture collapse of 3 or more ribs is 12-fold higher [17]. On the basis of our results, we suggest that for patients with serious fractures (e.g. flail chest), the posterior rib collapse and lateral fractures of the 6th to 8th ribs should be fixed first to ensure the stability of the thoracic structure, which can reduce the complications caused by the volume reduction and help patients improve their long-term respiratory function. However, fractures of the 4th and 5th ribs, especially on the left side, should be managed carefully because they may be associated with damage to the heart or important vascular structures; fractures of the 9th rib should be managed while considering the possibility of injuries to abdominal organs such as the liver and spleen [14].

To best of our knowledge, the methodology we applied in this study (computer simulation and stereo model, and a multiple variable linear regression analysis) is quite very novel, which provides a new idea for the similar research in the future. The strengths of this study are as follows. First, simulated rib fractures are free from the influence of pain, pulmonary contusion, and pulmonary infection, which will result in insufficient pulmonary ventilation in actual patients, and the chest volume obtained by CT is less than the actual value [18, 19]. Second, computer simulations of rib fractures simulate the fracture on the basis of the normal thoracic model. Thus, the thoracic volume before and after the simulated fracture can be calculated to determine the reduction in thoracic volume after the fracture. However, in real rib fractures, the normal thoracic volume value before the injury cannot be obtained. Third, computer modeling of the rib model is ideal and accurate, which can allow assessments of different degrees of displacement and collapse modes in the fracture model.

The limitations of this study are as follows. First, the computerized-modelled rib fracture collapse is obtained under ideal conditions, which is different from the actual clinical condition. This study aimed to find out which rib fracture displacement in the lateral rib fracture has the greatest influence on the thoracic volume by computer simulation, not to supply the clinical evidence. The simulated results need to be further verified in clinical practice. Second, we obtained good simulation results for lateral rib fractures in this study. However, we failed to simulate the anterior rib fracture model and posterior rib fractures.

## Conclusion

In general, the influence of rib fracture displacement and collapse on thoracic volume in fractures of the 6th through 8th ribs are critical in lateral rib fractures. For patients with 6th to 8th rib fractures and posterior rib collapse, operational decisions to perform surgical intervention to restore thoracic volume may be more important. For patients undergoing surgical treatment of flail chest, the collapse of the 6th to 8th ribs and posterior ribs should be first fixed while maintaining the stability of the thoracic cage.

## Supplementary information

**Additional file 1.** Multiple linear regression results for the three collapse modes.

## Data Availability

The datasets generated and analyzed during the current study are available from the corresponding author on reasonable request.

## Abbreviations

CBT: Chest blunt trauma
CXR: Chest X-ray
CT: Computed tomography
RCTs: Randomized controlled trials
