# Supplementary material for: Assessment of thoracic volume changes after the collapse of lateral rib fractures based on chest computed tomography data: computer simulation and a multiple variable linear regression analysis
Source: J Cardiothorac Surg. 2020 Jul 9;15:167. doi: 10.1186/s13019-020-01213-z (PMC7346514; doi:10.1186/s13019-020-01213-z)
Supplement: Supplementary file 1 — Additional file 1. Multiple linear regression results for the three collapse modes. [file 13019_2020_1213_MOESM1_ESM.doc]

**Appendix I.** Multiple linear regression results for the three collapse modes.


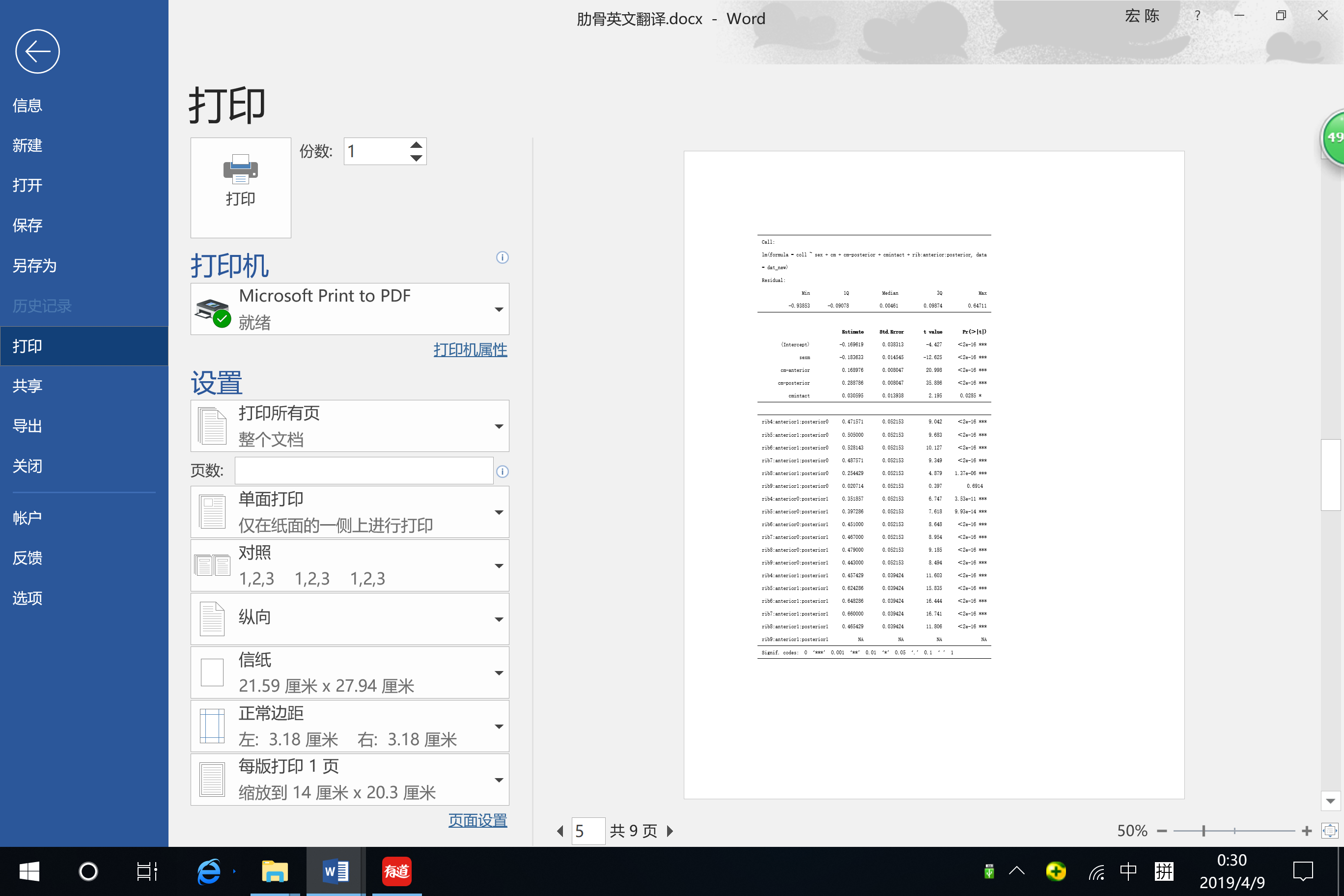


Anterior 1: posterior 0 = anterior collapse mode

Anterior 0: posterior 1 = posterior collapse mode

Anterior 1: posterior 1 = simultaneous anterior and posterior collapse mode
